# Xylooligosaccharide Modulates Gut Microbiota and Alleviates Colonic Inflammation Caused by High Fat Diet Induced Obesity

**DOI:** 10.3389/fphys.2019.01601

**Published:** 2020-01-22

**Authors:** Yanquan Fei, Yan Wang, Yilin Pang, Wenyan Wang, Dan Zhu, Meigui Xie, Shile Lan, Zheng Wang

**Affiliations:** College of Bioscience and Biotechnology, Hunan Agricultural University, Changsha, China

**Keywords:** obesity, high-fat diet, colon inflammation, gut microbiota, SCFA

## Abstract

Obesity leads to colonic inflammation and may increase the risk of colorectal cancer. Xylooligosaccharide (XOS) exhibits strong antioxidant and excellent antibacterial properties, and can be utilized by gut microbes to maintain the ecological balance of the intestinal tract. In this study, we explored how XOS modulates the microbiota and regulates high fat diet (HFD) induced inflammation. We measured the changes in body weight and visceral coefficients in rats fed a high-fat diet. We also measured the expression levels of inflammatory factors in the plasma and colonic tissues of the rats using the enzyme-linked immunosorbent assay and real-time quantitative polymerase chain reaction. We analyzed the composition of fecal microorganisms and short chain fatty acid (SCFA) content using 16S rDNA and GC-MS. We found that XOS significantly counteracted HFD induced weight gain. Moreover, the plasma levels of monocyte chemoattractant protein-1, tumor necrosis factor (TNF-α) and lipopolysaccharide decreased in the XOS-treated rats. XOS treatment decreased TNF-α mRNA expression and increased occludin mRNA expression in the rat colon. We observed a reduction in the overall microbial abundance in the feces of the XOS-treated rats, although the proportion of Bacteroidetes/Firmicutes increased significantly and the number of beneficial bacteria increased in the form of dominant microbes. We found that both SCFA-producing bacteria and SCFA content increased in the gut of the XOS-treated rats. We identified a correlation between the abundance of *Prevotella* and *Paraprevotella* and SCFA content. Taken together, we propose that XOS can alleviate colonic inflammation by regulating gut microbial composition and enhancing SCFA content in the gut.

## Introduction

Obesity is a global health problem (Cox et al., 2015), affecting nearly two billion people worldwide. Obesity is associated with high blood pressure, diabetes, and other chronic diseases. Recent studies have shown that obesity is also closely related to chronic inflammation (Liu et al., 2012). Excess fat deposits in the body induce intestinal structural and morphological changes, including damage to the colonic crypt and colonic epithelium, a reduced number of goblet cells and an increased number of intestinal epithelial cells (Paturi et al., 2010). Obesity also changes the composition of intestinal microbiota. The intestine is a complex micro-ecological system. The micro-ecological balance of the intestinal microflora and its metabolites are inextricably linked to human health (Lynch and Pedersen, 2016). The gut microbiota and certain intestinal microbial metabolites maintain the integrity of the intestinal barrier and epithelium, which are key to preventing high fat diet (HFD) induced colonic damage (Peng et al., 2009). Importantly, these microbes can digest carbohydrates to produce short chain fatty acids (SCFAs), which maintain intestinal health and relieve colonic inflammation. Moreover, SCFAs can supply energy to the intestinal cells and regulate their cell cycle progression (Pryde et al., 2002; Di Sabatino et al., 2005).

Xylooligosaccharide (XOS) is a sugar oligomer of 2–7 xylose single molecules linked by β-1,4 (Jordan and Kurt, 2010), a hydrolysate of dietary fiber commonly found in corn stover, wheat bran and rice bran (Ma et al., 2017). XOS shows strong antioxidant and antibacterial activity *in vitro* (Gao et al., 2012). Although XOS cannot be digested by humans, it can be metabolized by intestinal microbes (Gao et al., 2017). Indeed, XOS promotes the growth of *Bifidobacterium* and *Lactobacillus* in the gut and increases the SCFA content in these intestinal microbes, in turn enhancing the intestinal barrier function (Li et al., 2015). In pre-diabetic patients, XOS can enhance insulin sensitivity and reverse the changes in microbial composition caused by insulin insensitivity (Yang et al., 2015). Specifically, dietary supplementation of XOS can increase the relative abundance of *Lactobacillus* spp. and *Bifidobacterium* spp. in the gut, and the expression of tight junction protein occludin (OCLN) in the cecal tissues (Christensen et al., 2014). Both XOS and *Bifidobacterium* can enhance the immune function of the host (Childs et al., 2014). Studies have shown that XOS promotes the glycolysis of bifidobacteria throughout the gut, leading to an increase in SCFA concentration (Hansen et al., 2013). Here, we hypothesized that XOS can regulate the intestinal flora, increase SCFA levels and alleviate HFD-induced colonic inflammation in obese rats.

## Materials and Methods

### Animal Experimental Design and Sample Collection

All of the animal procedures were performed in accordance with the Guidelines for Care and Use of Laboratory Animals of Hunan Agricultural University. The protocol was approved by the Animal Care and Use Committee of Hunan Agricultural University. Thirty male Sprague Dawley rats (weighing 280 ± 20 g, aged 8 weeks, *n* = 30) were purchased from Hunan Silaike Jingda Co. (Changsha, China), with a certificate number of HNASLKJ2016-0002. After 1 week of acclimatization, the rats were randomly divided into three groups: normal control (NC) (*n* = 10), HFD (*n* = 10), and HFD plus xylooligosaccharide (HFD + XOS) (*n* = 10). The NC group was fed a normal diet (total calorie rate of 3.6 kcal/g and 72.3% of the caloric percentage of carbohydrates), and the HFD groups were fed a high fat diet (total calorie rate of 4.6 kcal/g, 46.4% of the caloric percentage of carbohydrates). After 7 weeks on the specified diets, the NC and HFD groups were given purified water by gavage, while the HFD + XOS group was given 2 g/kg XOS solution by gavage. XOS (CNS: Lu XK13-217-00581) of 95% purity was purchased from Shandong Longli Biotechnology Co. (Shandong, China). The animals were housed under cyclical illumination conditions (12-h light/dark cycle) with free access to food and water. They were weighed once a week. After 7 weeks of gavage, the rats were fasted for 12 h and sacrificed using pentobarbital sodium. The liver, heart, and spleen of each mouse were completely cut and weighed. At the same time, the white fat wrapped around the kidneys and the white fat attached to the testicles were cut and weighed. The colonic tissue was harvested and weighed. The middle part of the colon was fixed in 10% formalin, and the rest was preserved in liquid nitrogen. Heparin anticoagulant was added to the whole blood samples. The mixture was incubated for 30 min and spun at 12,000 rpm for 15 min at 4°C. The supernatant was then collected and stored.

### Histopathological Analysis

Colon and liver tissues from three group (*n* = 6) were removed from the fixative solution and slowly flushed with water. The tissue mass was then soaked in ethanol of different concentrations and dehydrated at 37–45°C for 2–4 h. Next, the tissue was embedded in paraffin wax (SVA, Uppsala, Sweden) and sectioned at a slice thickness of 5 μm. The sections were stained with hematoxylin and eosin, and imaged using a microscope (ML31, MSHOT, Guangzhou, China).

### Enzyme-Linked Immune Sorbent Assay

The content of lipopolysaccharide (LPS), interleukin 6 (IL-6), interleukin 10 (IL-10), tumor necrosis factor (TNF-α), and monocyte chemoattractant protein-1 (MCP-1) in the plasma from three group (*n* = 10) was measured using CUSABIO kits (CSB-E14247r/E04640r/E04595r/E11987r/E07429r, Wuhan, China) and a microplate reader (14041717, VT, United States).

### Real-Time Fluorescence-Based Quantitative PCR

Total RNA was extracted from the colonic tissues from three group (*n* = 10) using TriQuick Reagent (Solarbio, Beijing, China), and quantified and purified using an ultra-micro UV visible spectrophotometer (NanoDrop 2000, Thermo, United States). The RNA was reverse-transcribed into cDNA using a PrimeScript^TM^ RT reagent kit with gDNA Eraser (TaKaRa, Japan) and stored at −80°C. The resulting cDNA was analyzed by conducting the real-time quantitative polymerase chain reaction with a SuperReal PreMix Plus (SYBR Green) reagent kit (TIANGEN, Beijing, China) with various primers (Supplementary Table S1). The relative expressions were determined using the 2-ΔΔCt method.

### 16S rDNA and Illumina MiSeq Sequencing

The colonic contents from three group (*n* = 5) were collected in a sterile sampling tube, frozen in liquid nitrogen and extracted using a DNA extraction kit (Majorbio Bio-Pharm Technology, Shanghai, China). The extracted DNA was detected using 1% agarose gel electrophoresis to ensure the purity and integrity of the DNA. Qualifying samples were used to construct the library. The Amplicon fragment of interest was recovered, and the sticky ends formed by the disruption were repaired into blunt ends using T4 DNA Polymerase, Klenow DNA Polymerase and T4 PNK. By adding the base “A” at the 3′ end, either the DNA fragment was ligated to a specific linker with a “T” base at the 3′ end, or a double-index fusion primer containing a sequencing linker was designed and synthesized using genomic DNA as a template. Fusion primer PCR, magnetic bead screening purpose Amplicon tablets Section, finally, cluster preparation and sequencing were performed using a qualified library. The data obtained were used for the corresponding biological information analysis. All of the offline data were analyzed by the Beijing Genomics Institute).

### SCFA Content in Feces

Sample preparation: Rat feces from three group (*n* = 5) were collected after gavage. ddH_2_O was added and the samples were mixed, incubated at 4°C overnight and centrifuged. The supernatant was mixed with 25% metaphosphoric acid (Sinopharm, Shanghai, China) at a volume ratio of 1:1, incubated at room temperature for 4 h, and centrifuged at 12,000 rpm for 15 min. The supernatant was filtered using a 45-μm microporous filtration membrane, and the SCFA content was determined using gas chromatography–mass spectrometry (GC-MS) (Agilent 7890-5975C, Santa Clara, CA, United States).

Standard curve: A stock solution of SCFAs (Sigma, St. Louis, MO, United States) was prepared and preserved at 4°C (avoiding light). The stock solution was prepared as a standard solution based on the sample concentration before the measurement.

Chromatographic condition: Chromatographic analysis was conducted using DB-FFAP of 30 m × 250 μm × 0.25 μm equipped with a flame ionization detector (FID). The flow rate of high-purity nitrogen was 0.8 mL/min. The auxiliary gas was hydrogen with a high purity. The injection port and FID detector temperature were 250 and 280°C, respectively. The sample injection volume in the GC-MS analysis was 1 μL. The initial temperature was 60°C, increasing by 20°C/min to 220°C for 1 min.

### Statistical Analysis

All of the data were generated using SPSS 16.0 software and are represented here as mean ± standard deviation (SD). Differences between the mean values were evaluated using one-way analysis of variance and Tukey’s multiple comparisons test (if applicable). A *P* value < 0.05 was considered to be statistically significant.

## Results

### Physical Characteristics

**FIGURE 1:**
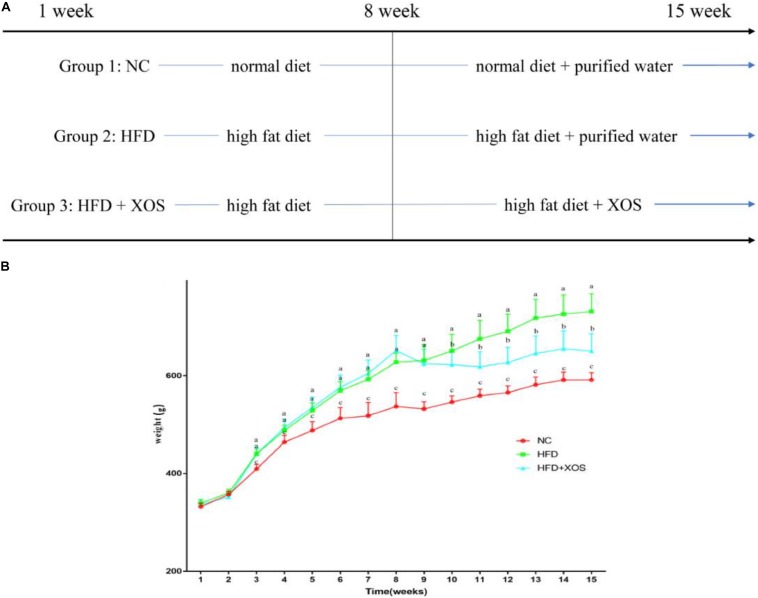
Effects of HFD and XOS on body weight of rats. **(A)** The process of treatment, **(B)** body weight changes. Data are given as mean ± SD (*n* = 10), ^a, b, c^mean values with different letters are significantly different from each other (*P* < 0.05).

**FIGURE 2:**
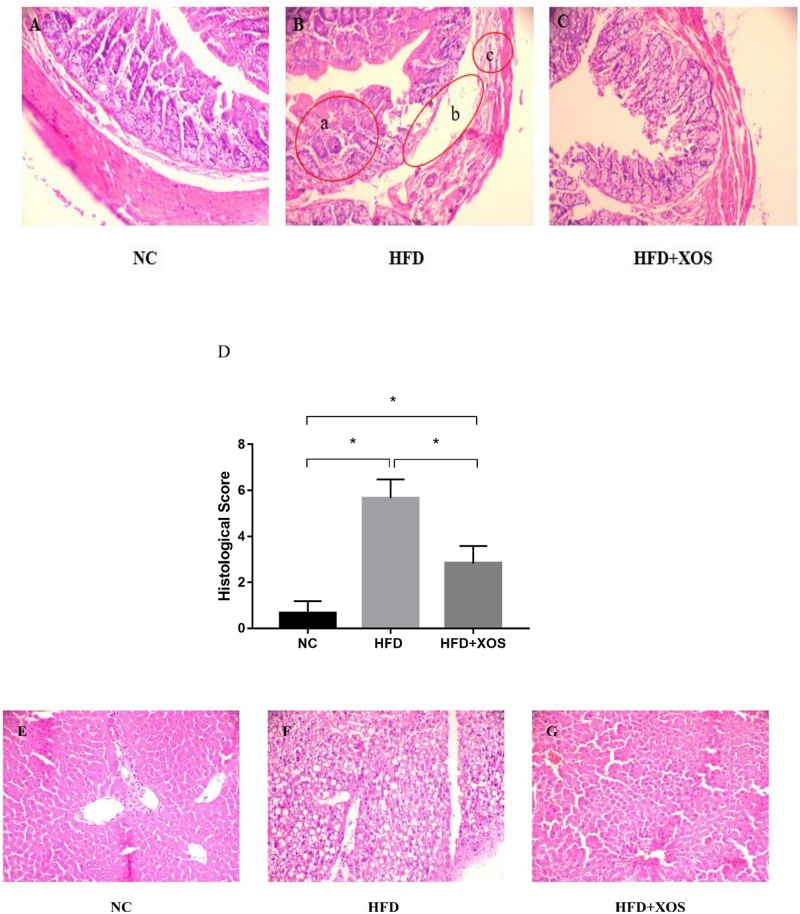
Representative colon HE staining results. The colonic tissues of NC rats **(A)**, HFD rats **(B)**, (**a**: crypt injury, **b**: intestinal edema of the colonic mucosa, and **c**: intestinal edema of the colonic mucosa), and HFD + XOS rats **(C)** were imaged at 20× magnification. **(D)** Histological score. The liver tissues of NC rats **(E)**, HFD rats **(F)**, and HFD + XOS rats **(G)** were imaged at 20× magnification. Data are given as mean ± SD (*n* = 10), *P < 0.05.

The body weights of the HFD and HFD + XOS rats were significantly greater than those of the NC rats from week 3 onward (Figure 1). The difference in body weights between the HFD and NC rats was 20% by the end of week 7. XOS was administered to the HFD + XOS rats from week 8 onward. Compared with the HFD rats, the HFD + XOS rats weighed significantly less from week 10 onward, despite being significantly heavier than the NC rats (Figure 1). The effects of XOS treatment on the body weight, organ weight and organ/body weight ratios of the rats are shown in Table 1. The body weight of the HFD rats was significantly greater than that of the NC rats. XOS treatment partially inhibited body weight gain in the HFD rats. These results indicated that XOS counteracted the effects of the HFD. The weight of the liver, perirenal fat and epididymal fat, and their corresponding organ/body weight ratios, were significantly higher in the HFD rats than the NC rats. However, these effects were largely inhibited in the HFD + XOS rats. Moreover, the HFD + XOS rats and the NC rats exhibited similar liver weight and liver/body weight ratios. There were no significant weight differences in the heart and spleen between the three groups of rats, and their corresponding organ/body weight ratios were also similar. We performed a morphological analysis of the middle segment of each group (*n* = 6). We used the histologic scoring system to analyze the morphology of the colon (*n* = 6) (Table 2). In the morphological analysis (Figure 2), we observed intact crypt structures and no significant inflammatory infiltration in the intestinal epithelial surface of the colonic tissues of the NC rats. In contrast, intestinal edema of the colonic mucosa (Figure 2Bb), severe crypt injury (Figure 2Ba), fracture on the surface of the colon (Figure 2Bc), loss of goblet cells and significant inflammatory infiltration were found in the HFD rats. Importantly, the HFD + XOS rats exhibited less severe colonic tissue damage, more intact crypt structures and reduced inflammatory infiltration compared with the HFD rats. In the liver morphology analysis, we can find that compared with the NC group, the liver of the HFD group showed obvious fat accumulation vacuoles, and macrophages and neutrophils aggregated, and there was obvious inflammatory infiltration. However, after treatment with XOS, these levels of inflammatory infiltration have been alleviated.

**TABLE 1:** Organ/body weight ratio.

Physique-related indicators	NC	HFD	HFD + XOS
Body weight (g)	591.3 ± 22.67^c^	728.20 ± 52.4^a^	655.00 ± 51.62^b^
Liver (g)	16.01 ± 3.72^b^	24.97 ± 3.70^a^	19.91 ± 5.20^b^
Organ/body weight ratio (%)	2.52 ± 0.21^b^	3.49 ± 0.24^a^	2.74 ± 0.30^b^
Perirenal fat (g)	9.57 ± 2.32^c^	24.5 ± 6.01^a^	13.99 ± 2.92^b^
Organ/body weight ratio (%)	1.6 ± 0.36^c^	3.36 ± 0.0047^a^	2.21 ± 0.32^b^
Epididymal fat (g)	8.61 ± 2.53^c^	20.89 ± 3.77^a^	14.01 ± 3.05^b^
Organ/body weight ratio (%)	1.45 ± 0.24^c^	2.76 ± 0.28^a^	2.33 ± 0.2^b^
Heart (g)	1.57 ± 0.18	1.61 ± 0.17	1.53 ± 0.20
Organ/body weight ratio (%)	0.26 ± 0.02	0.23 ± 0.01	0.23 ± 0.02
Spleen (g)	0.85 ± 0.18	0.95 ± 0.16	0.87 ± 0.12
Organ/body weight ratio (%)	0.15 ± 0.03	0.13 ± 0.02	0.13 ± 0.02

*Organ/body weight ratio (%) is the percentage of the weight of the corresponding organ. Data are given as mean ± SD (*n* = 10), ^*a, b, c*^mean values with different letters are significantly different from each other (*P* < 0.05).*

**TABLE 2:** The histologic scoring system.

Inflammation	Crypt Injury	Ulceration	Score
No significant inflammation	No injury	No ulceration	0
Neutrophilic inflammation in epithelium or lamina propria	Loss of basal one third of crypts	Two or fewer foci of ulceration	1
Inflammatory cells extending into submucosa	Loss of basal two thirds of crypts	Three or four foci of ulceration	2
Transmural inflammation	Loss of full thickness crypts	Diffuse/confluent ulceration	3

### Plasma Inflammatory Cytokines

**FIGURE 3:**
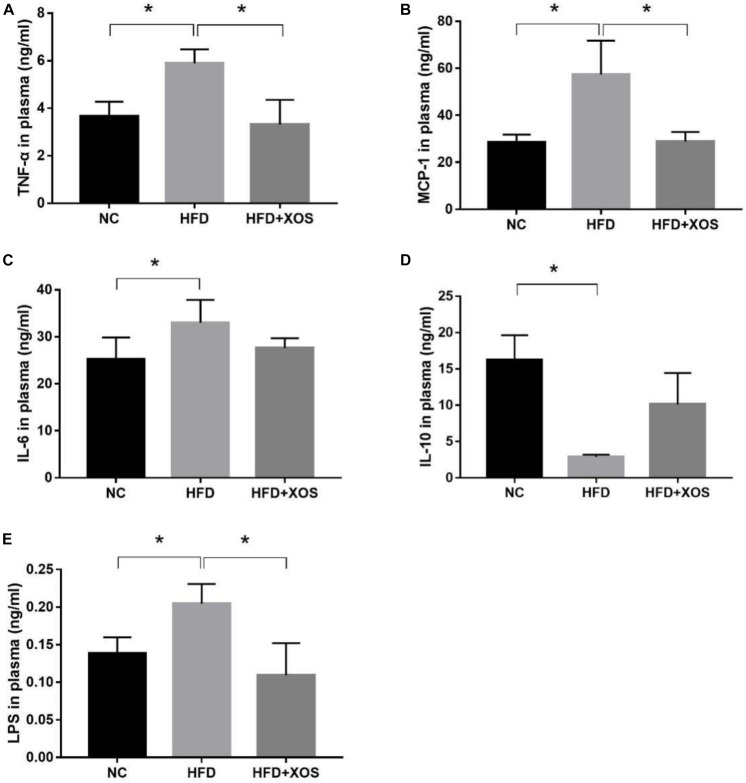
Impact of XOS treatment on the expression of plasma inflammatory cytokines. The levels of TNF-α **(A)**, MCP-1 **(B)**, IL-6 **(C)**, IL-10 **(D)**, and LPS **(E)** were determined. Data are given as mean ± SD (*n* = 10), *P < 0.05.

We suspected that long-term HFD could lead to colonic inflammation in rats. We therefore determined the levels of inflammatory cytokines in the plasma of our three groups of rats. As shown in Figure 3, the levels of TNF-α, MCP-1, IL-6 and LPS were significantly higher in the HFD rats than the NC rats. In contrast, the levels of TNF-α, MCP-1 and LPS were similar between the HFD + XOS rats and the NC rats. IL-6 levels were similar between the three groups of rats. IL-10 levels were significantly lower in the HFD rats than in the NC rats, and XOS supplementation reversed this trend.

### Colonic Inflammatory Cytokines

**FIGURE 4:**
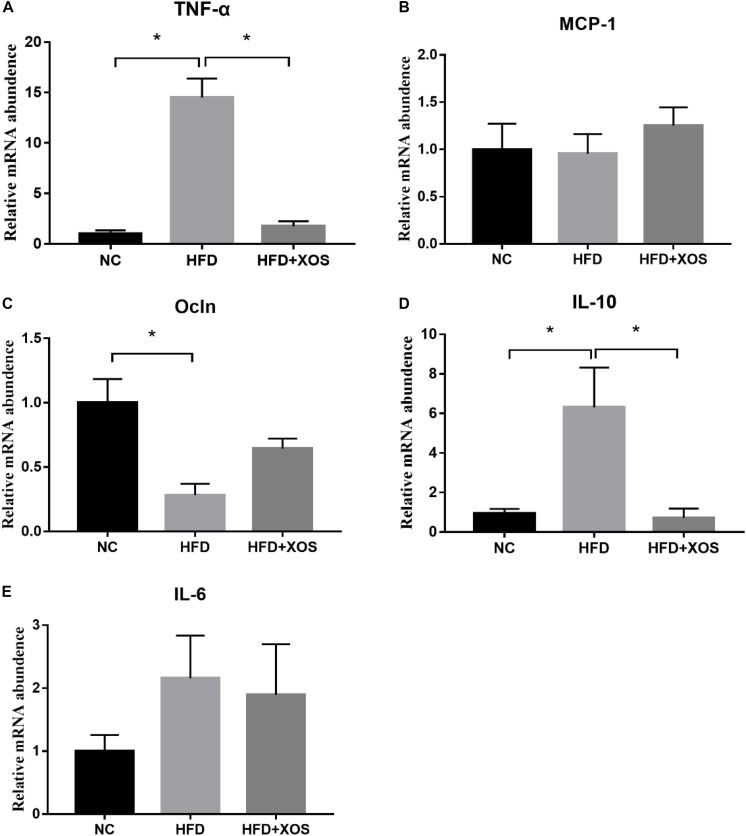
Impact of XOS treatment on the expression of colonic inflammatory cytokines. The relative gene expression levels for TNF-α **(A)**, MCP-1 **(B)**, OCLN **(C)**, IL-10 **(D)**, and IL-6 **(E)** were determined using RT-PCR. Data are given as mean ± SD (*n* = 10), *P < 0.05.

We next measured the levels of expression of inflammatory factors and tight junction proteins in the colonic tissues of the rats. As shown in Figure 4, we found significantly higher levels of colonic TNF-α expression and IL-10 mRNA expression in the HFD rats than the NC rats. In contrast, colonic TNF-α expression and IL-10 mRNA expression were similar between the HFD + XOS and NC rats. Moreover, colonic OCLN mRNA expression was significantly lower in the HFD rats than the NC rats. However, we found no significant differences in the colonic MCP-1 expression and IL-6 mRNA expression between the three groups.

### Rat Fecal Microbe Composition

**FIGURE 5:**
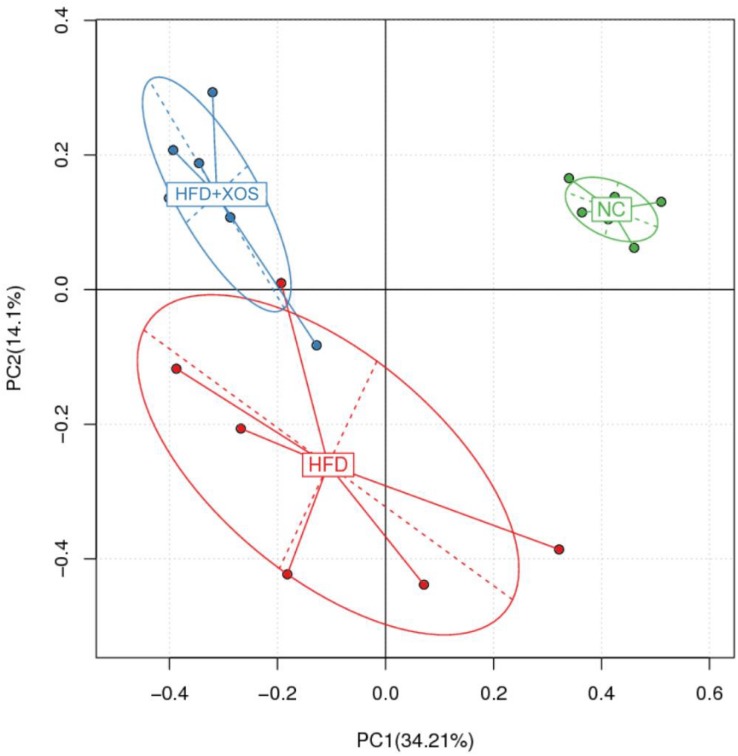
Principal component analysis (PCA) score plot based on the OTU abundance. NC, normal control group; HFD, high fat diet group; HFD + XOS, XOS plus high fat diet (*n* = 5).

The intestinal microflora plays an important role in obesity and lipid metabolism. We thus examined the intestinal microbial components in the three groups of rats by measuring fecal 16srRNAs. We obtained 103,259 optimized sequences in each sample (*n* = 5). We then clustered these optimized sequences to obtain the number of operational taxonomic units (OTUs) in each sample. As shown in Table 3, the HFD rats had significantly fewer OTUs in the feces than the NC rats. Interestingly, the number of OTUs in the feces was lower for the HFD + XOS rats than the other two groups. First, we performed the determination of α-diversity. Fecal microbial diversity was significantly lower in the HFD rats than in the NC rats, as indicated by the Sobs, Chao, Ace and Shannon indices. Surprisingly, fecal microbial diversity was significantly lower in the HFD + XOS rats than in the other two groups. This may have been due to the favorable effects of XOS on certain species of microbes associated with lipid metabolism or inflammatory responses. The addition of XOS resulted in a significant increase in the percentage of SCFA-producing bacteria such as *Prevotella*, while the percentage of pathogens such as *Oscillospira* was significantly reduced. At the same time, *Prevotella* became the absolute dominant flora in the gut. To analyze the overall difference in β-diversity, principal component analysis (PCA) was performed on all of the samples (Figure 5). We observed significant microbial community structure clustering in our three groups of rats.

**TABLE 3:** Alpha-diversity analysis.

	NC	HFD	HFD + XOS
OTU	854.00 ± 66.63^a^	602.83 ± 148.01^b^	422.50 ± 49.51^c^
Sobs	854 ± 0.0432^a^	602.83 ± 0.0422^b^	422.5 ± 0.0657^c^
Chao	956.12 ± 72.31196^a^	706.71 ± 144.64124^b^	487.98 ± 50.55842^c^
ACE	942.72 ± 68.42615^a^	688.13 ± 147.20679^b^	480.65 ± 49.31497^c^
Shannon	4.3074 ± 0.08499^a^	4.1574 ± 0.43369^a^	3.6343 ± 0.127^b^
Simpson	0.0432 ± 0.01153^b^	0.0422 ± 0.01686^b^	0.0657 ± 0.0165^a^

*Results of experiments using the mixed linear regression model for analysis of independent effects of sample type on baseline sample alpha-diversity. Data are given as mean ± SD (*n* = 5), ^*a, b, c*^mean values with different letters are significantly different from each other (*P* < 0.05).*

**FIGURE 6:**
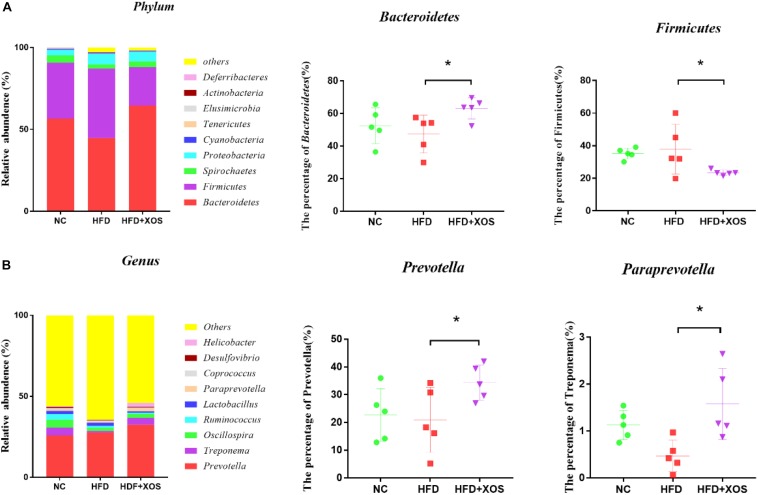
Microbial composition analysis. Taxonomic composition of the fecal bacterial communities at the phylum level **(A)**. Taxonomic composition of the fecal bacterial communities at the genus level **(B)**. Data are given as mean ± SD (*n* = 5), *P < 0.05.

At the phylum level (Figure 6A), *Bacteroidetes*, *Firmicutes*, *Spirochaetes*, and *Proteobacteria* had the highest phase abundances and were the dominant bacteria in the three groups of rats. The relative abundances of *Bacteroidetes*, *Firmicutes*, *Spirochaetes*, and *Proteobacteria* in the NC rats were 56.53, 34.21, 4.57, and 3.33%, respectively. The relative abundances of *Bacteroidetes*, *Firmicutes*, *Spirochaetes*, and *Proteobacteria* in the HFD rats were 44.81, 42.32, 2.59, and 6.54%, respectively. The relative abundances of *Bacteroidetes*, *Firmicutes*, *Spirochaetes*, and *Proteobacteria* in the HFD + XOS rats were 64.56, 23.41, 3.52, and 6.01%, respectively. Compared with the HFD rats, HFD + XOS rats exhibited an increased percentage of *Bacteroidetes* (*P* < 0.05) and a decreased percentage of *Firmicutes* (*P* < 0.05).

At the genus level (Figure 6B), *Prevotella*, *Treponema*, and *Oscillospira* were the most abundant microorganisms in the three groups of rats. The relative abundances of *Prevotella*, *Treponema*, and *Oscillospira* in the NC rats were 25.91, 7.35, and 4.92%, respectively. The relative abundances of *Prevotella*, *Treponema*, and *Oscillospira* in the HFD rats were 23.88, 2.31, and 4.63%, respectively. The relative abundances of *Prevotella*, *Treponema*, and *Oscillospira* in the HFD + XOS rats were 32.46, 4.23, and 2.56%, respectively. The relative abundances of *Prevotella* and *Paraprevotella* were significantly higher in the HFD + XOS rats than the NC rats (*P* < 0.05).

### Fecal SCFA Content and Its Association With Microbial Composition

**FIGURE 7:**
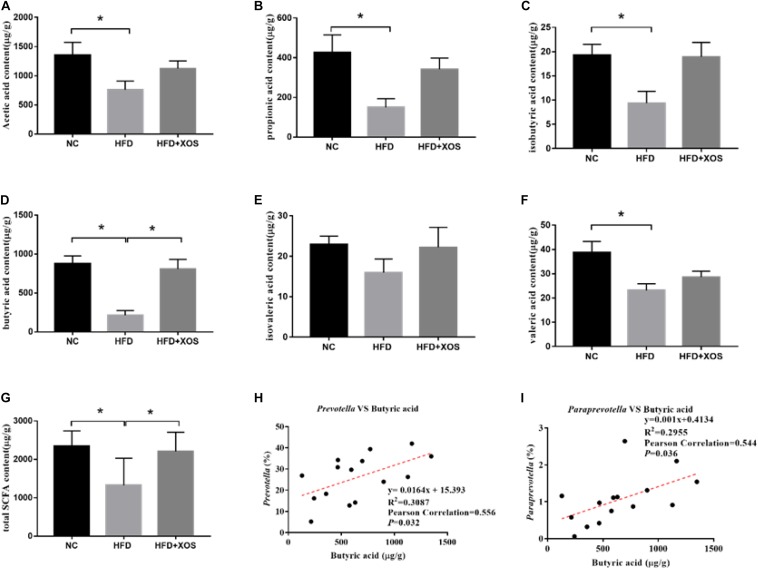
Determination of fecal SCFA content and its correlation with abundance of microorganisms. The levels of acetic acid **(A)**, propionic acid **(B)**, isobutyric acid **(C)**, butyric acid **(D)**, isovaleric acid **(E)**, and valeric acid **(F)** were determined. The correlation between *Prevotella* and acetic acid **(G)**, the correlation between *Bacteroidetes* and acetic acid **(H)** and the correlation between *Lactobacillus* and acetic acid **(I)** were analyzed. Data are given as mean ± SD (*n* = 5), *P < 0.05.

To verify whether the anti-inflammatory effects were a direct consequence of compositional changes in the intestinal microbiota or an indirect effect involving metabolites, we examined the fecal SCFA content in the three groups of rats (Figures 7A–G). The levels of acetic acid, propionic acid, butyric acid, isobutyric acid, valeric acid, and total SCFAs were found to be significantly lower in the HFD rats than in the NC rats. The levels of acetic acid, propionic acid, isobutyric acid, and valeric acid were similar between the three groups. The levels of butyric acid and total SCFAs in the HFD + XOS rats were significantly higher than those in the HFD rats and similar to those in the NC rats. The levels of fecal isovaleric acid were similar between the three groups. Previous reports have suggested that the SCFA content is regulated by microorganisms. Therefore, we analyzed the correlation between the percentage of intestinal microbes and the SCFA contents of all samples (Figures 7H,I). We found a positive correlation between butyric acid and the relative abundances of *Prevotella* and *Paraprevotella.*

## Discussion

Dietary supplementation of XOS has been found to alleviate intestinal inflammation (Yoshino et al., 2006). XOS can modulate inflammatory cytokines such as TNF-α and IL-1β in mice (Hansen et al., 2013). Here, we successfully modeled obesity and systemic low-grade inflammation in rats using HFD. Specifically, the rats fed a HFD exhibited significant increases in body weight, organ fat deposition, organ weight and the organ/body weight ratio. The HFD rats also exhibited structural damage to the intestine. We found that XOS effectively alleviated many of the adverse effects of HFD, including body and organ weight gain, as well as some systemic chronic low-grade inflammation. Long-term HFD can also lead to colonic damage, which is characterized by the shortening of the colon, marked damage to the crypts and the disappearance of goblet cells (Deol et al., 2015).

We speculate that inflammatory damages are due to the high level of LPS. LPS is a lipopolysaccharide produced by Gram-negative bacteria, and can cause inflammation via TLR4-mediated NF-kB activation (Hoshino et al., 1999) and the production of various inflammatory factors, such as TNF-α, IL-6, and IL-1β (Dinarello, 1991). In our experiments, the morphological damage to the colon was relieved by XOS, and the plasma LPS content and expression of TNF-α and MCP-1 were reduced. Our results showed that plasma and colonic IL-10 levels exhibited similar trends in the NC and HFD-XOS rats. In contrast, the HFD rats exhibited increased colonic IL-10 levels and decreased plasma IL-10 levels. There are two possible reasons for this reciprocal behavior. First, IL-10 is produced by a variety of immune cell types, including monocytes and macrophages, which are potent inhibitors of pro-inflammatory cytokines and chemokines, and can prevent diet-induced insulin resistance (Akdis and Blaser, 2001; Hong et al., 2009). Plasma IL-10 can prevent insulin resistance, and the level of blood glucose in the blood is constantly changing. We measured the level of plasma IL-10 at one time point. As the blood glucose level decreases after fasting, and the role of IL-10 in preventing diet-induced insulin resistance weakens, the IL-10 level is low. Second, we measured IL-10 protein levels in the plasma and IL-10 mRNA levels in the colon. In addition, previous studies have shown that XOS can improve intestinal health by regulating intestinal microbes (Makelainen et al., 2010), which may also contribute to the reduction of colonic inflammation observed in the HFD-XOS rats compared with the HFD rats.

Intestinal microbes are intimately linked with the immune system of the host (Zhu et al., 2017; Metzger et al., 2018). There is a mutually beneficial relationship between the microorganisms and the host. The metabolites of some microorganisms are beneficial to the physiological functions of the host, while the host provides energy for the growth and activity of the microorganisms (Kamada et al., 2013; Zhou et al., 2017). The disruption of the balance between the host and gut microbes is associated with many diseases (Littman and Pamer, 2011). Intestinal microbes can increase intestinal permeability and thus control obesity-induced inflammation (Cani et al., 2008). We thus analyzed the composition of intestinal microbes in the three groups of rats. Previous studies have shown that HFD causes tremendous changes in the gut microbial composition in mice (Turnbaugh and Gordon, 2009). Indeed, an increase in Firmicutes and a decrease in Bacteroidetes are common trends in both obese humans and obese mice (Ley et al., 2005; Moschen et al., 2012). Our results showed that the Firmicutes/Bacteroidetes ratio nearly doubled in the HFD rats compared with the NC rats, and that this effect was inhibited by XOS treatment. At the genus level, XOS treatment led to a significant increase in the percentages of *Prevotella* and *Paraprevotella*. *Prevotella* is a Gram-negative anaerobic bacterium that helps break down proteins and carbohydrates. *Prevotella* abundance is thought to be associated with polysaccharides and fiber-rich plant-based diets (Gorvitovskaia et al., 2016; Ley, 2016). One study showed that metformin improves inflammation in type 2 diabetic rats by selectively acting on *Prevotella* and reducing IL-6 and TNF-α levels (Liu et al., 2018). In patients with clinical inflammatory bowel disease (IBD), *Prevotella* abundance at the site of inflammation is significantly reduced (Hirano et al., 2018).

Related studies have shown that *Prevotella* can synthesize SCFAs using pyruvate as a substrate via the acetyl-CoA pathway (Rey et al., 2010; Louis et al., 2014). We suspected that XOS treatment altered the composition of gut microbes and promoted SCFAs production in rats. SCFAs are thought to be involved in lipid metabolism and transport (Marcil et al., 2002). Although the specific mechanism by which SCFAs are involved in lipid metabolism is unclear, SCFAs promote lipid oxidation in tissues and reduce the storage of white fat (den Besten et al., 2013). SCFAs increase the expression of PGC-1α and AMPK phosphorylation to promote lipid oxidation in tissues (Donohoe et al., 2011). Additionally, SCFAs are metabolites that can improve lipid metabolism and enhance immune functions. Studies have shown that SCFA treatment increases the expression of antimicrobial peptides such as LL-37 and CAP-18 in human intestinal epithelial cells (Raqib et al., 2006); promotes the expression of IL-18, which is a cytokine that maintains the internal stability of the intestinal epithelium; and acts on the epithelial barrier itself (Kelly et al., 2015).

Both *Prevotella* and *Paraprevotella* are butyrate-producing bacteria (Gao et al., 2018; Kong et al., 2019). We showed that the total levels of SCFAs and butyric acid were significantly increased after XOS treatment in rats. Moreover, we found that the abundance of *Prevotella* and *Paraprevotella* positively correlated with the levels of butyric acid. These results indicated that XOS regulated the composition of intestinal microbes and increased the abundance of butyric acid-producing bacteria such as *Prevotella* and *Paraprevotella*. In the MetS, CRC, Colitis, and other model experiments, the increase of Fecal butyrate content will reduce the expression of TNF-α, IL-2, NF-κB, and other pro-inflammatory factors (Rodriguez-Cabezas et al., 2002; Hijova et al., 2013; Hald et al., 2016). Studies have shown that butyric acid can inhibit the expression of PPARγ, thereby alleviating many PPARγ-related diseases including IBD (Kinoshita et al., 2002; Alex et al., 2013). As butyric acid is a known to inhibit inflammation (Manrique Vergara and Gonzalez Sanchez, 2017), increased butyric acid content can be assumed to alleviate obesity-induced inflammation.

Studies have shown that the composition of intestinal microflora in humans and rats is similar, and it is mainly composed of *Firmicutes* and *Bacteroidetes* at the level of the phylum. However, the proportion of *Prevotellaceae* and *Prevotella* in the human intestinal flora is reduced compared with rats. On the other hand, the individual differences of human intestinal microbes are also significantly higher than that of rats, which may be related to the diet and lifestyle of human subjects. As we know, diet is one of the most important elements in shaping gut microbes. Therefore, we are convinced that the dietary supplement of XOS can change the intestinal microbial structure of humans and increase the bacteria which can produce butyric acid to improve human intestinal immunity.

## Conclusion

Our study revealed that XOS alleviated obesity-led intestinal inflammation. We also found that XOS treatment increased the proportion of butyric acid producing bacteria in the rat intestine. Taken together, these results indicate that XOS protects against obesity-induced colonic inflammation by improving the intestinal microbial structure and increasing the abundance of SCFA-producing microbes.

## Data Availability Statement

The datasets generated for this study can be found in NCBI SRA https://www.ncbi.nlm.nih.gov/sra/?term=PRJNA593411.

## Ethics Statement

All animal procedures were performed in accordance with the Guidelines for Care and Use of Laboratory Animals of Hunan Agricultural University.

## Author Contributions

YF and YW performed the study and conducted the data analysis. YP, SL, and ZW designed the research. WW, DZ, and MX provided the assistance for the study. YF and ZW prepared the first draft of the manuscript. All authors read and revised the manuscript.

## Conflict of Interest

The authors declare that the research was conducted in the absence of any commercial or financial relationships that could be construed as a potential conflict of interest.
