# Supplementary material for: Xylooligosaccharide Modulates Gut Microbiota and Alleviates Colonic Inflammation Caused by High Fat Diet Induced Obesity
Source: Front Physiol. 2020 Jan 22;10:1601. doi: 10.3389/fphys.2019.01601 (PMC6987399; doi:10.3389/fphys.2019.01601)
Supplement: Supplementary file 1 [file Table_1.docx]

**Supplementary Table S1** | Primer sequences used for real-time PCR.

| Gene | Primer sequence （ 5′-3′） |
| --- | --- |
| *TNF-α*  *IL-6*  *MCP-1*  *IL-10*  *Occludin(Ocln)*  *β-Actin* | F: CCACGCTCTTCTGTCTACTG  R: GCTACGGGCTTGTCACTC  F: CCTTCTTGGGACTGATGT  R: CTCTGGCTTTGTCTTTCT  F: AATGAGTCGGCTGGAGAA  R: GCTTGAGGTGGTTGTGGA  F: GCTATGTTGCCTGCTCTT  R: ATGCTCCTTGATTTCTGG  F: CAGACCACTATGAAACCGACTA  R: TCTCCAGCAACCAGCATC  F: TGACAGGATGCAGAAGGAGA  R: TAGAGCCACCAATCCACACA |
